# Nudging Healthy Choices in Food Search Through Visual Attractiveness

**DOI:** 10.3389/frai.2021.621743

**Published:** 2021-04-22

**Authors:** Alain D. Starke, Martijn C. Willemsen, Christoph Trattner

**Affiliations:** ^1^ Marketing and Consumer Behaviour Group, Wageningen University and Research, Wageningen, Netherlands; ^2^ Department of Information Science and Media Studies, University of Bergen, Bergen, Norway; ^3^ Department of Industrial Engineering and Innovation Sciences, Eindhoven University of Technology, Eindhoven, Netherlands; ^4^ Recommender Lab, Jheronimus Academy of Data Science, 's-Hertogenbosch, Netherlands

**Keywords:** recipes, nudging, food health, food search, information retrieval

## Abstract

Recipe websites are becoming increasingly popular to support people in their home cooking. However, most of these websites prioritize popular recipes, which tend to be unhealthy. Drawing upon research on visual biases and nudges, this paper investigates whether healthy food choices can be supported in food search by depicting attractive images alongside recipes, as well as by re-ranking search results on health. After modelling the visual attractiveness of recipe images, we asked 239 users to search for specific online recipes and to select those they liked the most. Our analyses revealed that users tended to choose a healthier recipe if a visually attractive image was depicted alongside it, as well as if it was listed at the top of a list of search results. Even though less popular recipes were promoted this way, it did not come at the cost of a user’s level of satisfaction.

## 1 Introduction

Individual food choices are fundamental in the prevention of obesity and its health consequences (Chan and Woo, 2010). However, the World Health Organization states that the full effect of one’s individual responsibility can only be achieved when people have access to a healthy lifestyle (WHO, 2018), which should be both enabled and supported by the environment in which food choices are made (Furst et al., 1996).

AI has the potential to provide such support to its users (Freyne and Berkovsky, 2010; Schäfer et al., 2017). Many of today’s ‘food interactions’ are online (Trattner and Elsweiler, 2017a), often at recipes websites that allow users to explore new foods to cook at home. AI is used to optimize the relevance of the presented content through search and retrieval algorithms, which typically lead the promotion of popular recipes to all users (Trattner et al., 2018). Unfortunately, popular recipes on websites tend to be unhealthy (Trattner et al., 2017), which raises a challenge: how can AI present healthier content to users, while maintaining relevance?

To this end, Information Retrieval (IR) studies describe two main routes as to how users find relevant food or recipes. This either involves search queries (e.g., in search boxes) or personalized food recommendations (e.g., “more like this” suggestions). Most food search research seeks to improve how relevant content is retrieved in response to keywords or other inputs (El-Dosuky et al., 2012; Helmy et al., 2015; Carvalho et al., 2018), as well as how to optimize auto-completion queries and responses to questions (Cunningham and Bainbridge, 2013). However, only incremental steps have been taken to improve the healthiness of retrieved recipes (van Pinxteren et al., 2011). In particular, there has not been much attention for how search results e.g., recipes are presented or explained to a user (Demarque et al., 2015; Mirsch et al., 2017; Grebitus et al., 2020).

As the other ‘main route’ to find relevant recipes, food recommender research focuses on personalizing content without using search queries (Trattner and Elsweiler, 2017a). For example, recipe websites present content that is similar to what a user has liked in the past, based on ratings and bookmarks (Trattner and Elsweiler, 2017a). However, recipes with many bookmarks also tend to have higher fat and calorie contents (Trattner et al., 2017). As a result, users of recipe websites are typically exposed to unhealthy recommendations that merely satisfy their short-term “cravings”. In other words, historical user preferences are unlikely to lead to healthy recommendations. Although recent recommender studies have shown possibilities to optimize for a recipe’s health (Ge et al., 2015; Musto et al., 2020), their focus to solve this problem through algorithmic development does not fully capture the complexity of food decision-making (cf. Furst et al. (1996)).

We argue that a different perspective is required to advance the health of users. While studies on food and AI have focused on changing what is recommended, we propose to adapt how food and recipes are presented, by changing the decision context (Johnson et al., 2012). Work by Elsweiler et al. (2017) provides evidence for this, showing that a user’s food preferences are affected by the visual attractiveness of the presented recipe. However, their findings have yet to be applied in an online food search context, since a feature-based approach to model visual attractiveness is missing (Khosla et al., 2014; Elsweiler et al., 2017). In a similar vein, various studies have used nudges in ‘offline’ contexts (e.g., presentation order effects in cafeteria (Thaler and Sunstein, 2009)), but much less is known about the effectiveness of ‘digital nudges’ to support healthy food choices (Mirsch et al., 2017). Moreover, it is unclear whether such nudges can be applied without impacting a user’s evaluation of a recipe website. For example, if a recipe website makes it harder for users to find popular recipes harder by prioritizing healthy recipes, it could negatively affect a user’s satisfaction with that website (West et al., 2013; Said and Bellogín, 2014).

In this study, we show that nudges can be instrumental in search-related food choices. We draw upon insights from both AI-related studies and social sciences to examine whether changing how search results are presented can persuade users to seek out healthier recipes. In particular, we use findings from Elsweiler et al. (2017) on the visual representation of recipes, as well as work from Carney and Banaji (2012), Bar-Hillel (2015) on the ranking of search results. We posit the following research question:

[RQ]: To what extent can visual enhancement of images and re-ranking of search results on a recipe website support healthy food choices, without decreasing user satisfaction?

In doing so, this paper presents two main contributions:

1. The first user study in the context of food search that shows and models how healthy food choices can be promoted without decreasing user satisfaction, by adapting the visual attractiveness of presented recipe images and re-ranking search results on their healthiness.
2. A preliminary study in which we model the perceived attractiveness of images that accompany a recipe using image features (e.g., brightness, colorfulness).

### 1.1 Related Work

Food preferences are the result of a context-dependent, multi-faceted process (Furst et al., 1996; Palojoki and Tuomi-Gröhn, 2001). Hence, to address our research question, we discuss related work from multiple streams of research. First, we highlight efforts in Information Retrieval (IR) and Recommender Systems research. Thereafter, we explain how insights from offline field studies in behavioral economics (i.e., nudging) can be translated to an AI context.

#### 1.1.1 Food Search

It is commonplace to look for online recipes through a web search engine (e.g., Google) or a recipe website’s own search framework (Svensson et al., 2000). However, recipe retrieval can only be successful if recipes are classified in terms of their attributes, which can be a challenging task if it involves multimodal content (Min et al., 2016). For example, recipes usually not only comprise a title and a list of ingredients (Van Erp et al., 2020), but also directions, images (Elsweiler et al., 2017), and sometimes even videos (Min et al., 2016; Chen et al., 2017). As a result, a considerable proportion of research is devoted to optimizing the retrieval of recipes, particularly in terms of relevance and efficiency (Helmy et al., 2015; Min et al., 2016; Trattner and Elsweiler, 2017a; Yang et al., 2017).

Less attention is devoted to how to capture a recipe’s healthiness. A few approaches show how to retrieve recipes in response to a query that explicitly mentions health (West et al., 2013; Helmy et al., 2015). Other approaches go beyond explicit search queries. For example, it has been suggested how to retrieve healthier replacements for relevant recipes, based on inter-recipe similarity (van Pinxteren et al., 2011; Asano and Biermann, 2019). However, most IR approaches seek to optimize relevance by ranking possible search results on their popularity, which often leads to unhealthy results (Trattner et al., 2017). Work on how to present search results of food or recipes is missing from the corpus of research.

#### 1.1.2 Food Recommender Systems

In a similar vein, food recommender systems focus on the question of ‘what to present?’ They retrieve relevant recipes based on what a user has liked in the past, which is optimized during and after each session (Trattner and Elsweiler, 2017a). As such information may be absent on recipe websites, a common strategy is to promote popular recipes, which unfortunately tend to be unhealthy (Trattner and Elsweiler, 2017b; Trattner et al., 2017). In fact, recommenders have devoted little attention to the healthiness of online recipes (Trattner et al., 2017), but new efforts are arguing for ‘health-aware’ recommender systems (Ge et al., 2015; Schäfer et al., 2017). What could be achieved is shown by a recent knowledge-based recommender study, which suggests that a combination of recipe features (e.g., fat content) and user features (e.g., eating goals) determine user preferences for healthier foods in a recommender (Musto et al., 2020).

However, food recommender studies only optimize the algorithm component of their systems. Studies in other domains (e.g., sustainability, social networks) show how explaining recommendations in terms of contextual factors (e.g., social norms) can steer user decision-making, such as by highlighting how many other users have selected a certain option (Sharma and Cosley, 2013; Starke et al., 2020). At the same time, such principles seem to be missing in the food AI domain, both in recommender systems and in food search (IR).

#### 1.1.3 Digital Nudges for Food Choices

Changing the decision context to affect behavior is investigated in behavioral economics. Such research refers to representation of a set of items as the choice architecture (Johnson et al., 2012). Specific aspects (e.g., social explanations) can be adapted in such an architecture to lead to predictable changes in the choice behavior of individuals, which is referred to as a ‘nudge’ (Thaler and Sunstein, 2009; Mirsch et al., 2017). In the context of food, supermarkets and cafeterias can re-arrange their product assortment (i.e., their choice architecture) to boost sales of specific products, such as by placing healthy foods at eye level (Thaler and Sunstein, 2009; Johnson et al., 2012). Another notable example is the use of nutrition labels (e.g., traffic light labeling), which indicates how healthy supermarket products or fast food menus are (Morley et al., 2013; Mohr et al., 2019; Niven et al., 2019).

A meta-analysis of healthy eating nudges shows that we can differentiate between three types (Cadario and Chandon, 2020). They are either cognitively oriented (e.g., influencing what consumers know through traffic light labels), affectively oriented (e.g., using photos to influence how consumers feel about a recipe), or behaviorally oriented (e.g., changing what people do by reorganization a product shelf). Cognitively oriented nudges were found to be the least effective, while affectively and behaviorally oriented nudges were found to be more effective. Between those two, affective factors (e.g., the taste of food) are generally found to be the strongest important determinant of food choices (Glanz et al., 1998; Januszewska et al., 2011).

#### 1.1.4 Affective Nudging Through Visual Bias

Online food studies also present evidence for the role of affect in food choices by examining images [cf. (Carvalho et al., 2018; Kitamura et al., 2009)]. An analysis of uploaded recipes on the social cooking website AllRecipes.com reveals that image features of a recipe photo (e.g., image sharpness, saturation) are related to its popularity (Trattner et al., 2018). This suggests that boosting the overall visual attractiveness of an image can also affect user preferences, and can be used as a type of nudge. Evidence for this is also presented in a study on biases in recipe choices (Elsweiler et al., 2017), revealing that visual attractiveness can nullify preferences for high-fat food (Elsweiler et al., 2017), which are typically popular on recipe websites (Trattner and Elsweiler, 2017b; Trattner et al., 2017). Elsweiler et al. (2017) presented pairs of comparable recipes to users that consisted of a low-fat and a high-fat option, but for which the low-fat recipe was predicted by a classification model to have a more attractive image. The low-fat recipe was chosen in 62.2% of the pairs, which showed that humans are biased towards attractive or tasty-looking images.

However, the findings in Elsweiler et al. (2017) have yet to be applied to a food search context. We expect that boosting the visual attractiveness of healthy alternatives in a list of recipes can serve as an affectively oriented nudge. Hence, in the current study, we will investigate whether showing attractive images alongside healthy recipes, as well as depicting less attractive images next to unhealthy recipes can persuade users to select healthier recipes. This leads to the following hypothesis:

[H1]: Users choose healthier recipes if healthy recipes are accompanied by a visually attractive image and unhealthy recipes are accompanied by a visually unattractive image.

#### 1.1.5 Presentation Order Effects

Another aspect in IR research is the order in which items are presented [cf. Jannach et al. (2010), Ricci et al. (2011)]. Lists of recipes, retrieved through either recommender algorithms or search queries, tend to present the Top-N most relevant recipes (Trattner and Elsweiler, 2017a). Whereas offline nudging studies have examined the order in which foods are presented in a cafeteria or supermarket (Thaler and Sunstein, 2009; Johnson et al., 2012), food AI studies often do not consider that presentation order effects within Top-N lists of recipes could further impact user choices (Trattner and Elsweiler, 2017a).

Literature on judgment and decision-making shows various presentation order effects. Items presented first in a list are more likely to be chosen (Mantonakis et al., 2009; Carney and Banaji, 2012), due to a primacy effect in item memorization. Other position effects are systematically reviewed in Bar-Hillel (2015) for different contexts (e.g., food choices in restaurants, purchases in web shops). Food choices are categorized as a decision context where items placed at either the beginning or the end of a list tend to be preferred, having a so-called ‘edge advantage’ (Bar-Hillel, 2015). Taken together, we expect that recipes presented first in a Top-N list of modest length, which does not trigger choice overload (Bollen et al., 2010; Scheibehenne et al., 2010), are more likely to be chosen than other recipes in a list.

Search results on recipe websites are typically sorted on their relevance. For example, AllRecipes.com sorts recipes by default on the match between the search query and the recipe’s description and name (i.e., ‘Best Match’) (Trattner and Elsweiler, 2017b), but this can be changed to ‘Popular’ or ‘Newest’. Since users prefer recipes that have high sugar or fat content (Power and Schulkin, 2013), the default presentation order prioritizes popular, yet unhealthy recipes (Trattner et al., 2017), which is thus reinforced by a primacy effect (Carney and Banaji, 2012; Bar-Hillel, 2015).

We expect that a presentation order effect can also be used to promote healthy recipes. Ordering recipe search results on their healthiness instead of their popularity could boost the selection of healthier options, due to the primacy effect. Hence, the current study investigates whether ranking recipes on their healthiness, instead of their popularity, positively affects user choices for healthy recipes, hypothesizing the following:

[H2]: Users choose healthier recipes from lists that are ranked on healthiness, instead of popularity.

#### 1.1.6 Combining Visual Attractiveness and Ranking

We expect that the merits of an increase in visual attractiveness and a presentation order are complementary. Preferences for visually attractive images stem from one’s emotions (Elsweiler et al., 2017), acting as an affectively oriented nudge (Cadario and Chandon, 2020). In contrast, re-ranking a list of recipes in terms of their health is a behaviorally oriented nudge (Bar-Hillel, 2015; Cadario and Chandon, 2020). Hence, we hypothesize that these two effects will lead to an additive effect when combined:

[H3]: Users choose healthier recipes if these are accompanied by a visually attractive image and are ranked at the top of the search result list, compared to lists that are subject to only one or none of these manipulations.

#### 1.1.7 User Evaluation of Food AI

A common metric to evaluate the effectiveness of both AI and nudging interventions is the chosen item, food, or recipe (Thaler and Sunstein, 2009; Trattner and Elsweiler, 2017a). However, users might evaluate an AI system more negatively if not relevance but another feature is prioritized, such as a recipe’s healthiness. Recommender system research underlines that behavioral data, such as choices, needs to be contextualized through a user’s evaluation (Knijnenburg and Willemsen, 2015; Ekstrand and Willemsen, 2016). For example, a study on a video recommender system shows that while users watched more videos in a non-personalized condition (Knijnenburg et al., 2010), their reported levels of perceived system effectiveness and satisfaction were higher in the personalized condition.

We expect that a user’s evaluation of a list of recipe search results consists of two components. First, we consider a user’s perceived list attractiveness (PLA), which is also used in Willemsen et al. (2016), to assess a user’s evaluation of the list of recipes. Second, we gauge a user’s choice satisfaction, which captures the user’s experience surrounding the chosen recipe (Knijnenburg and Willemsen, 2015; Willemsen et al., 2016; Starke et al., 2017). Willemsen et al. (2016) show that a user’s perceived list attractiveness positively affects choice satisfaction.

Evidence for a negative impact on the user’s evaluation due to digital food nudges is limited (Mirsch et al., 2017). However, this relation has been examined in the context of ‘offline’ restaurants and canteens (Kallbekken and Sælen, 2013; Bergeron et al., 2019; Saulais et al., 2019), where the implemented nudges lead to healthier food choices without affecting customer satisfaction. Although these only concerned behaviorally oriented nudges [cf. Cadario and Chandon (2020)], it is reasonable to expect that both our visual attractiveness and re-ranking manipulations will not negatively affect a user’s evaluation. Since the contents of the Top-N list are not changed but only the presentation order is, we expect no relation with the perceived list attractiveness. We hypothesize the following:

[H4]: Re-ranking recipe search results according to their healthiness does not affect the user’s perceived list attractiveness.

In a similar vein, we neither expect that adapting the visual attractiveness of recipe images according to their healthiness affects a user’s perceived list attractiveness. We hypothesize the following:

[H5]: Increasing the visual attractiveness of healthy recipe images (and vice versa for unhealthy recipes) will not affect a user’s perceived list attractiveness.

In the following sections, we present our food search study. In Section 2, we describe the used dataset, the selected recipes, how we modelled visual attractiveness in our preliminary study, and the methodology of the main user study. Thereafter, in Section 3, we report the analyses and main findings of our user study, while Section 4 discusses the implications of our results.

## 2 Materials and Methods

To be able to differentiate between healthy and unhealthy recipes, we compiled an appropriate subset. In addition, to discern between attractive and unattractive images (cf. [H1]), we applied a number of image modifications. Previous research suggests that popular images are characterized by specific features, such as their colors and texture (Khosla et al., 2014). Following this rationale, we performed a preliminary study in which the attractiveness of various images was rated by humans, after which it was modelled in terms of the underlying image features. Using this subset of recipes, we designed the methodology of our user study to examine whether visual attractiveness and health re-ranking could support healthy recipes choices.

### 2.1 Dataset

We used a dataset that contains recipes from AllRecipes.com, as also used by Elsweiler et al. (2017), Trattner et al. (2017), Trattner et al. (2018). Receiving an estimated 25 million unique visitors each month (EBizMBA, 2020), AllRecipes.com was the most popular recipe website on the internet in 2020. The dataset comprised 58,263 main dish recipes with various modalities, such as cooking directions, recipe ingredients, images, and popularity ratings. The current study used features regarding the visual attractiveness of a recipe, as well as its healthiness—as expressed by its nutritional content (i.e. fat, salt, sugar) and portion size. Additionally, we used a selection of low level features that described a recipe image’s properties (i.e., brightness, colorfulness, entropy, sharpness, saturation).^1^. These image features were included in the dataset, because they were calculated in Trattner et al. (2018). All features had values between 0 and 1, except Entropy.^2^, which ranged from 2.84 to 7.94 (*M* = 7.39, *SD* = 0.53).

### 2.2 Pre-Study on Visual Attractiveness

We performed a preliminary study, examining whether we could use image features to predict an image’s visual attractiveness. From the full dataset, a subset of 475 recipes was selected based on keyword matching for one out of four keywords: “Burger”, “Salad”, “Pasta”, and “Curry”. For each keyword, we selected the first 100 to 130 recipes that had all relevant metadata available (e.g., title, image, nutritional content).

All images were rated on their visual attractiveness. Five persons involved in the current research project (students and researchers) were asked “How visually attractive do you find this image?” for all 475 images. Each image’s visual attractiveness was rated on a 7-point scale, ranging from very unattractive (1) to very attractive (7).

To assess the reliability of these ratings, we computed the interrater agreement for each researcher pair. Since an exact match in ratings was unlikely due to the 7-point scale, we used decreasing scores of agreement for increasing differences between raters. For example, a difference of ‘0’ would score an agreement of ‘1’, a difference of ‘1’ scored ‘0.83’, ‘2’ led to ‘0.67’, etc. Using this method, we obtained a mean interrater agreement of 83.01% (*SD* = 1.46), which was considered reliable. The scores for each rater pair are outlined in Table 1.

**TABLE 1:** Interrater agreement per pair of raters.

	Rater 1 (%)	Rater 2 (%)	Rater 3 (%)	Rater 4 (%)	Rater 5 (%)
Rater 1	100				
Rater 2	82.35	100			
Rater 3	81.58	82.47	100		
Rater 4	83.82	83.39	79.86	100	
Rater 5	85.05	83.26	83.61	84.81	100

After normalizing the given ratings using standardized z-scores, we used linear regression to predict an image’s visual attractiveness using its features. Table 2 shows that four features positively affected visual attractiveness [*F*(5,450) = 45.67]: Brightness, Colorfulness, Entropy, and Sharpness. Saturation was found to negatively affect an image’s visual attractiveness. The model’s accuracy was reasonable, for it explained 29.06% of the variance.

**TABLE 2:** Robust linear regression predicting the visual attractiveness of a recipe image in terms its low level features. All features ranged between 0 and 1, except for Entropy (M=7.39, SD=0.53).

	*β*	S.E.	95%−CI
Brightness	3.22***	0.54	[2.16;4.28]
Colorfulness	8.35***	1.02	[6.35;10.35]
Entropy	0.80***	0.13	[0.54;1.05]
Sharpness	1.42**	0.42	[0.61;2.24]
Saturation	−4.08***	0.75	[−5.54;−2.62]
Constant	−6.53***	1.02	[−8.54;−4.52]
$R^{2}$	0.291***		

***p < 0.001, **p < 0.01, *p < 0.05.

### 2.3 Recipe Selection

For our main study, we sampled a total of 32 recipes from the selection used in our pre-study. We used four different search queries (i.e., “burger”, “salad”, “pasta”, “curry”), which were all main dishes. To answer our research question, we sought to compile a set of recipes that were similar on most attributes, but which varied in terms health and image attractiveness.

#### 2.3.1 Recipe Healthiness (FSA Score)

**FIGURE 1:**
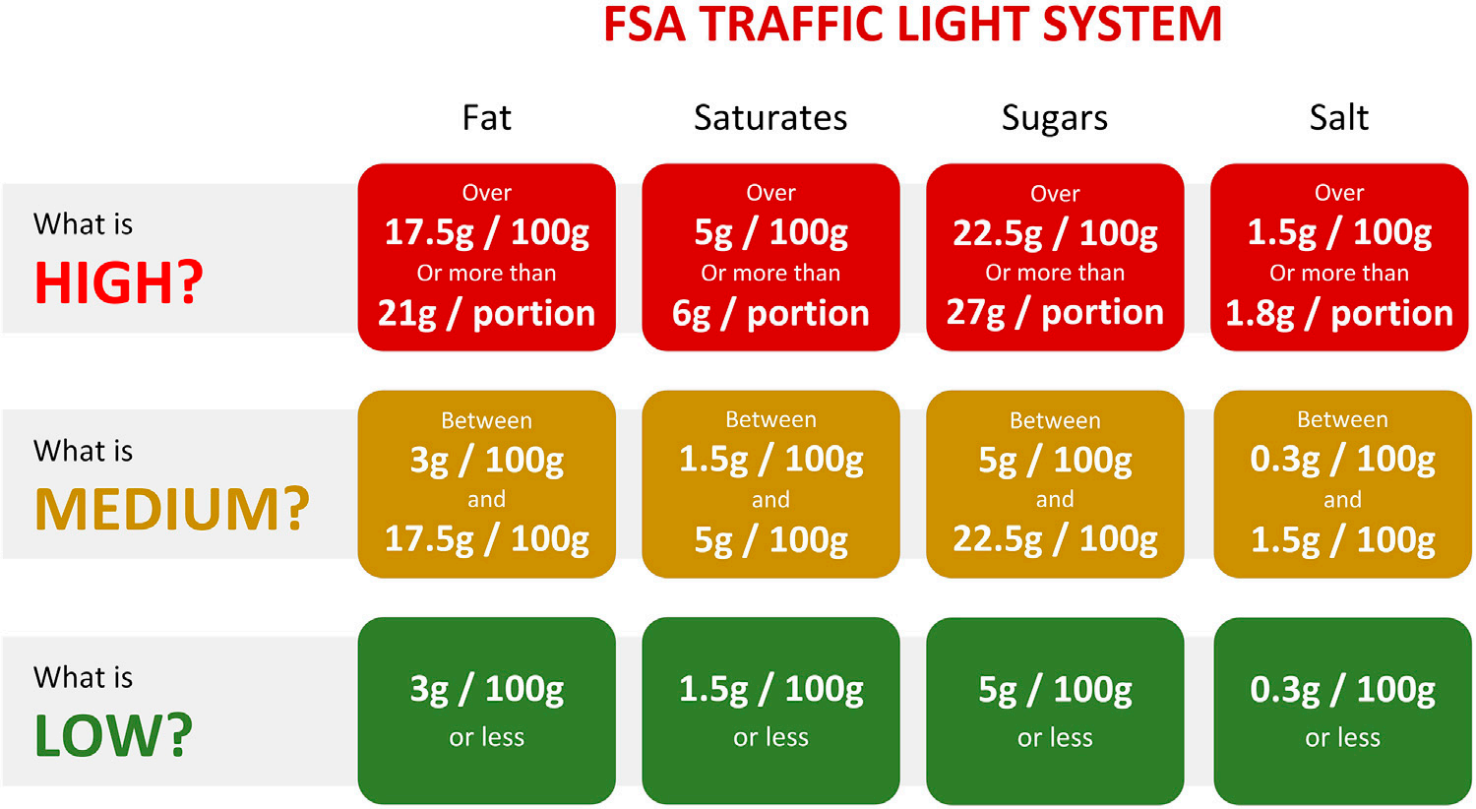
Depiction of the traffic light system (cf. of Health UK and Agency (2016), which was used to assess the healthiness of a recipe. This system was operationalized in an FSA score, ranging from 4 (i.e., all nutrients scored low; healthy) to 12 (i.e., all nutrients scored high; unhealthy).

We used the “traffic light” system of the UK Food Standards Agency to compute a recipe’s healthiness (of Health UK and Agency, 2016), also known as the ‘FSA score’. Figure 1 depicts how it was computed, scaling three macronutrients (i.e., sugar, fat, and saturated fat) and salt from green (healthy) to red (unhealthy). In line with Trattner et al (2017), we scored each recipe per nutrient (e.g., low in sugar, medium in fat, etc.) and computed a single FSA health score. Scores of ‘1’, ‘2’, and ‘3’ were given to the colors green, amber, and red, respectively, resulting in total scores between 4 (i.e., ‘all low’) and 12 (i.e., ‘all high’). In other words, a recipe with an FSA score of 4 was considered to be very healthy, while a score of 12 was seen as very unhealthy.

Eventually, we compiled sets of eight recipes that consisted of four healthy and four unhealthy recipes. Table 3 provides an overview of the selected recipes and their attributes. Curry, Salad, and Pasta recipes were considered to be ‘healthy’ if they had an FSA score of 6 or lower, and ‘unhealthy’ if they scored 9 or higher. Since most Burger recipes were rather unhealthy, we considered FSA scores of 7 or lower to be healthy for Burgers, while scores of 9 or higher were classified as unhealthy.

**TABLE 3:** Recipes used in the main study.

Recipe name	FSA	Fat	Sat. Fat	Sugar	Salt	Mean rating	Image attract.
Backyard black bean burgers	5	1.29	0.22	2.05	1.27	4	1.44
Burgers chili dog style	9	9.75	4.60	1.79	1.12	4.67	0.09
Butter bean burgers	7	11.90	3.31	1.07	0.78	4.38	–0.33
Cajun burgers	9	17.20	6.05	0.70	1.07	3.5	0.99
Carrot burgers	7	14.37	2.37	4.62	0.94	4.4	0.43
Cheddar bacon burgers	9	12.16	5.24	1.78	1.44	4.55	0.00
Pizza burgers	9	6.36	2.64	3.72	0.96	3	1.18
Pumpkin bean burgers	6	7.72	1.26	2.73	0.45	4	1.54
Curried chicken and potatoes	6	4.76	1.07	2.07	0.37	4.13	0.57
Curry chicken and brown rice casserole	6	0.81	0.20	5.88	0.63	4.29	0.18
Curry chicken with rice	10	13.28	3.26	7.11	0.12	4	–0.70
Curry mango chicken	9	6.87	2.80	4.10	0.33	4	–0.37
Curry sausage couscous	9	15.46	5.03	0.67	0.71	5	0.52
Curry-coconut shrimp	5	3.12	1.08	1.43	0.23	4.05	–0.02
Turkey curry	5	5.87	1.37	1.09	0.10	3.5	–0.67
Turkey curry with cashews	9	10.25	2.59	1.19	0.44	4	0.70
Fusili pasta with broccoli	5	0.78	0.12	5.64	0.03	4.5	1.56
Pasta pomodoro	5	5.67	0.90	2.01	0.27	4.53	1.11
Pasta sauce with Italian sausage	9	8.76	3.03	2.10	1.35	4.7	1.43
Pasta with chicken and roasted pepper cream sauce	9	10.54	5.86	1.37	1.14	4	0.27
Pasta with fresh vegetables	9	9.12	1.62	3.05	1.75	4.63	0.98
Pasta with lentil soup sauce	5	0.90	0.16	0.79	0.33	5	–0.53
Pasta with Salami and Peas	9	17.68	5.78	1.98	1.12	4	–0.46
Pasta with spinach and smoked sausage	5	2.54	0.57	1.64	0.50	4.75	0.13
Caesar salad with cilantro and green chile dressing	9	27.26	5.06	1.49	1.01	4.5	0.13
Cucumber-carrot salad	5	0.77	0.11	3.67	0.47	4.29	0.55
Curried cashew, pear, and grape salad	10	19.67	3.97	8.14	0.99	4.92	0.71
Shaved asparagus salad	6	7.59	1.40	1.28	0.55	5	0.13
Tomato bacon salad	10	11.81	3.27	1.44	1.24	0	1.54
Tomato, basil, and corn salad with apple cider dressing	5	6.37	0.87	1.57	0.22	5	1.51
Tomato-basil salad	5	5.23	1.35	2.64	0.18	5	0.99
Tricolore salad of endive, beet, and arugula, pantzaria salata	9	39.31	6.32	2.53	1.02	4.67	0.96

Recipes are clustered per type (e.g. Burger, Curry) and ordered alphabetically. The FSA score scale ranges from 4 to 12, where 4 is healthiest. Nutritional content is expressed per 100g, ratings run from 1 to 5 (0 indicates that no ratings had been given), and image attractiveness are standardized z-scores (used in the baseline condition).

#### 2.3.2 Image Modifications

Table 3 shows there were no large differences in the standardized image attractiveness of the selected recipes. To investigate whether healthy recipes accompanied by attractive images were selected more often than unhealthy recipes with unattractive photos, we used the regression model reported in Table 2 to modify the presented images.

**FIGURE 2:**
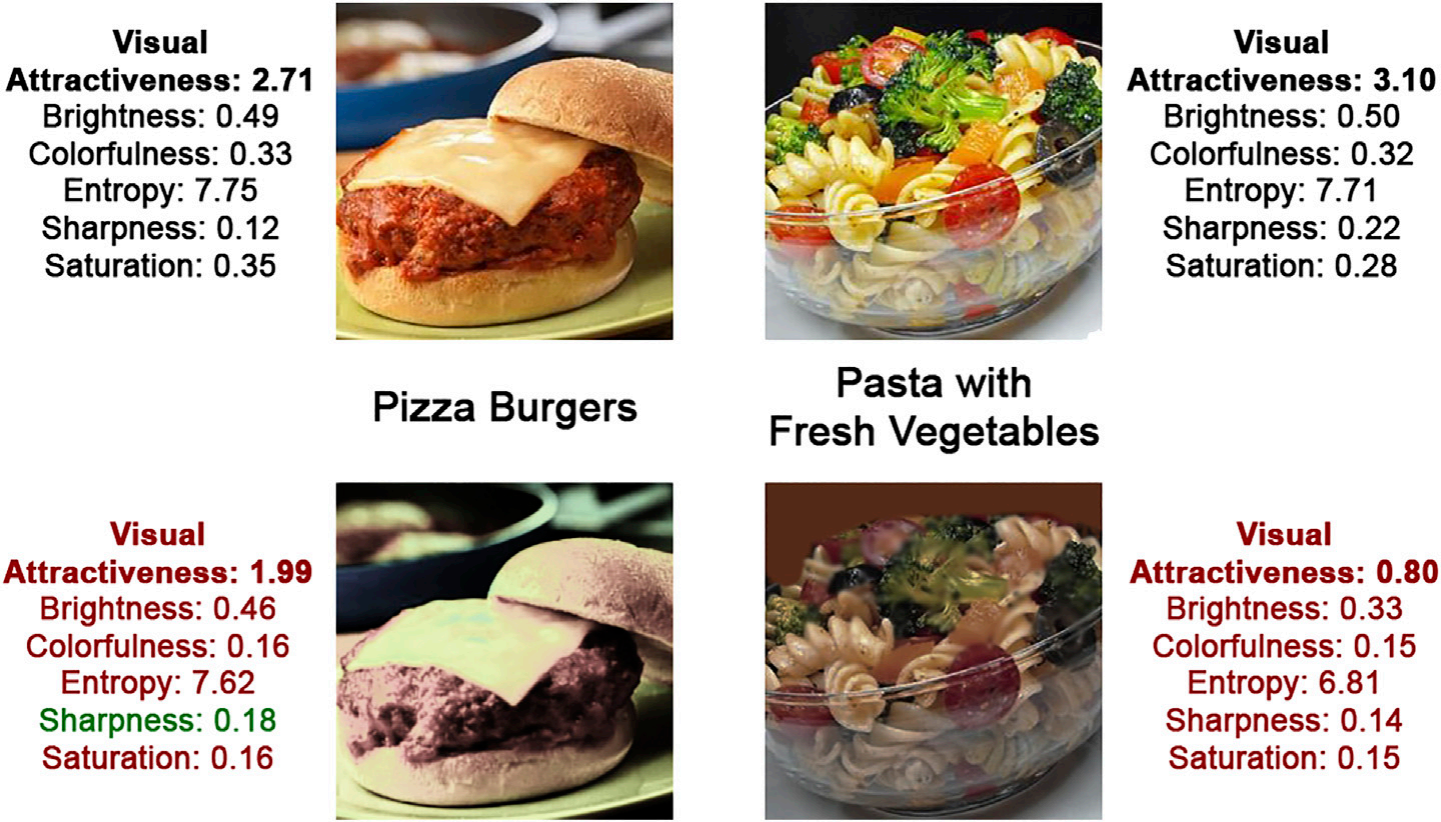
Example images that show how the attractiveness of database images (top row) was decreased (bottom row). The top row images were used in the non-visual conditions, the bottom images in the visual manipulation conditions. Depicted alongside a photo are its name and image attributes, which show that the predicted visual attractiveness decreased for both images, mostly due to a reduction in brightness and colorfulness. Note: All used images are available in our online repository: https://github.com/alainstarke/RecipeSearch.git.

On the one hand, images of unhealthy recipes were made less attractive. We used Adobe Photoshop 2019 to adapt one or more image features. Which features were modified and by how much was image-dependent, as we wanted each image to still appear as realistically as possible—which was judged by the researchers. For example, for one image, we reduced the image brightness and adapted the colorfulness to make the image seem more yellow, while for another image the saturation was increased sharply. Examples of both the original images (used in the baseline visual conditions) and the manipulated images (used in the visual manipulation conditions) are depicted in Figure 2. Consistent with the methods applied in Trattner et al. (2018), we computed the image features of the ‘downgraded’ substitutes and predicted the image attractiveness using the regression model described in Table 2. The image attractiveness of unhealthy recipes (i.e., scaled from 1 to 7) dropped on average by 0.95 (*SD* = 0.71), resulting in a mean predicted attractiveness of 1.42 (*SD* = 0.54).

**FIGURE 3:**
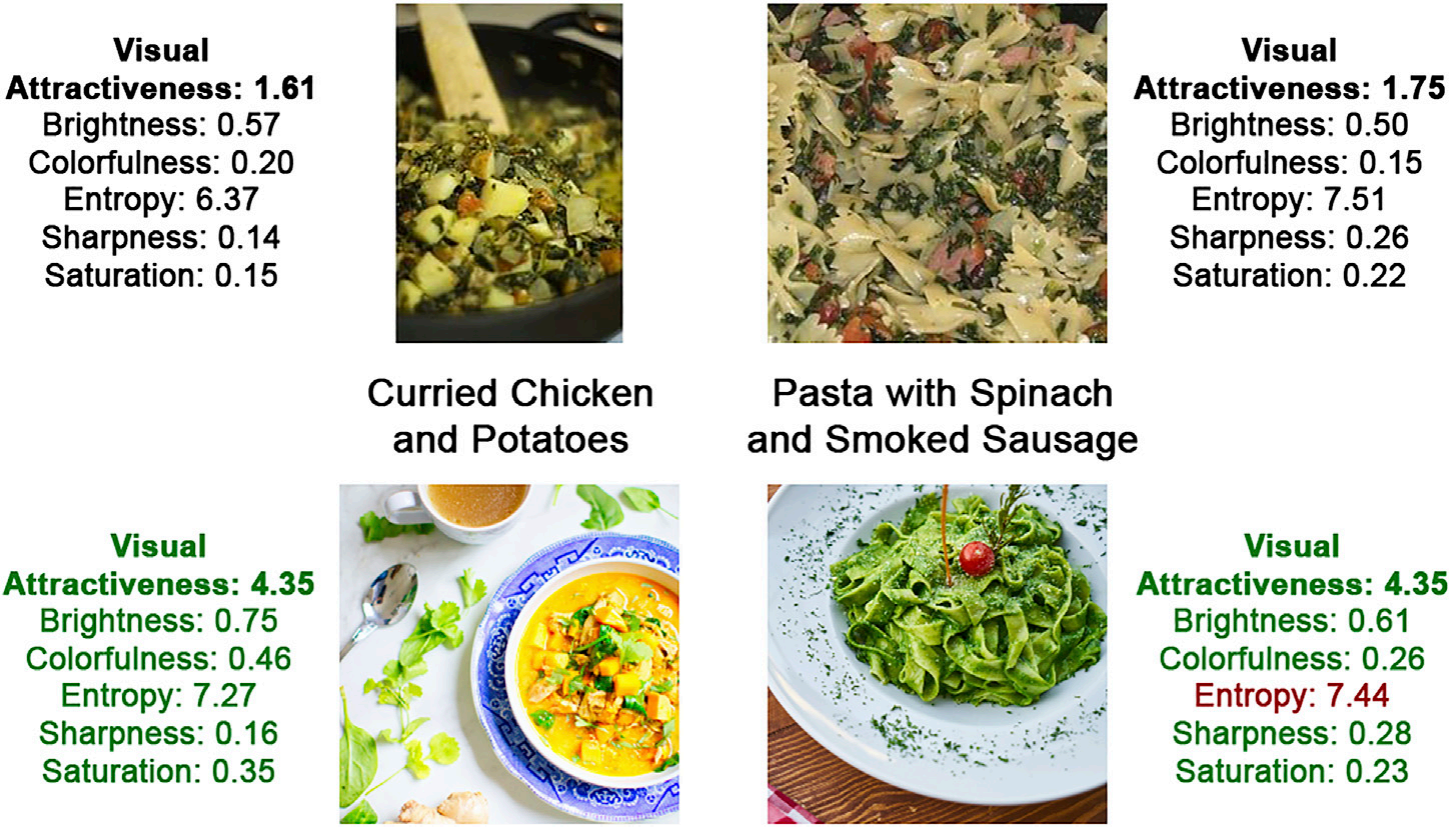
Examples of how original database images (top row) were replaced with more attractive, professional photos (bottom row), taken from unsplash.com. The top row images were used in the baseline visual conditions, the bottom images in the visual manipulation conditions. Depicted alongside a photo are its name and image attributes, which show that the predicted visual attractiveness improved for both images, mostly based on an increase in brightness and colorfulness. Note: All used images are available in our online repository: https://github.com/alainstarke/RecipeSearch.git.

On the other hand, we replaced ten images of healthy recipes with more attractive alternatives. Images were replaced if their standardized *z*-score in Table 3 fell below 0.99, or within 1 standard deviation above the mean. Two examples of the original images (used in the visual baseline conditions) and their replacements (used in the visual manipulation conditions) are depicted in Figure 3. New images were retrieved from the free-for-use online stock photo library unsplash.com, which offered high quality photos, often shot by professional photographers. Our search contained the recipe’s title as keywords, after which we required selected images to be similar to the original database image, as well as to depict most of the recipe’s ingredients. Using the approach of Trattner et al. (2018), we again computed the image features of the substitute images and predicted the image attractiveness using the regression model described in Table 2. The image attractiveness of healthy recipes increased on average by 1.06 (*SD* = 0.76). Combined with the six unchanged images, we found a mean predicted image attractiveness of 3.00 (*SD* = 0.65) for healthy recipes, which was 1.56 higher than the mean attractiveness of unhealthy recipes.

### 2.4 Search Prototype

For the purpose of our main study, we implemented a search prototype with common auto completion functionalities. As the backend for our search prototype, we relied on Apache Lucence.^3^, which was regarded as the state-of-the-art in search implementations, comparable to the technology of Google. The autocompletion mechanism was implemented with the help of the well-known typeahead.js framework.^4^, an open-source framework developed by Twitter.

**FIGURE 4:**
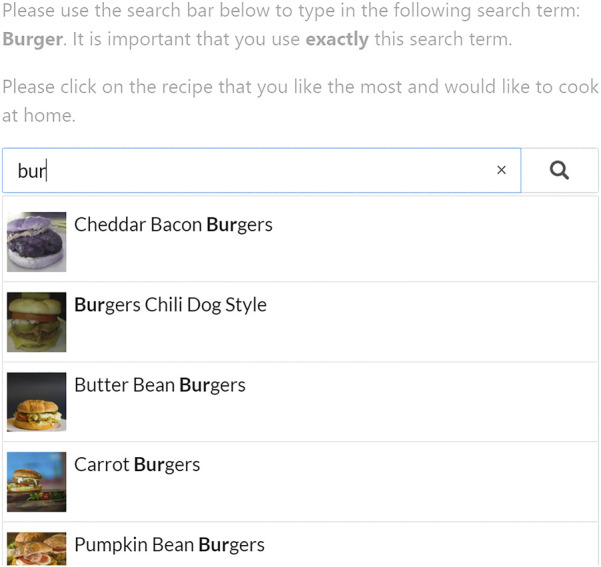
Screenshot of the search prototype interface, including the user study’s instructions at the top.

Our search prototype is depicted in Figure 4. Users could search for recipes by submitting a specific keyword in the search bar. In return, the search tool presented eight recipes in a vertical item list below the search bar, displaying the title of the recipe and its depiction, but omitting further details.

### 2.5 User Study: Research Design and Procedure

**FIGURE 5:**
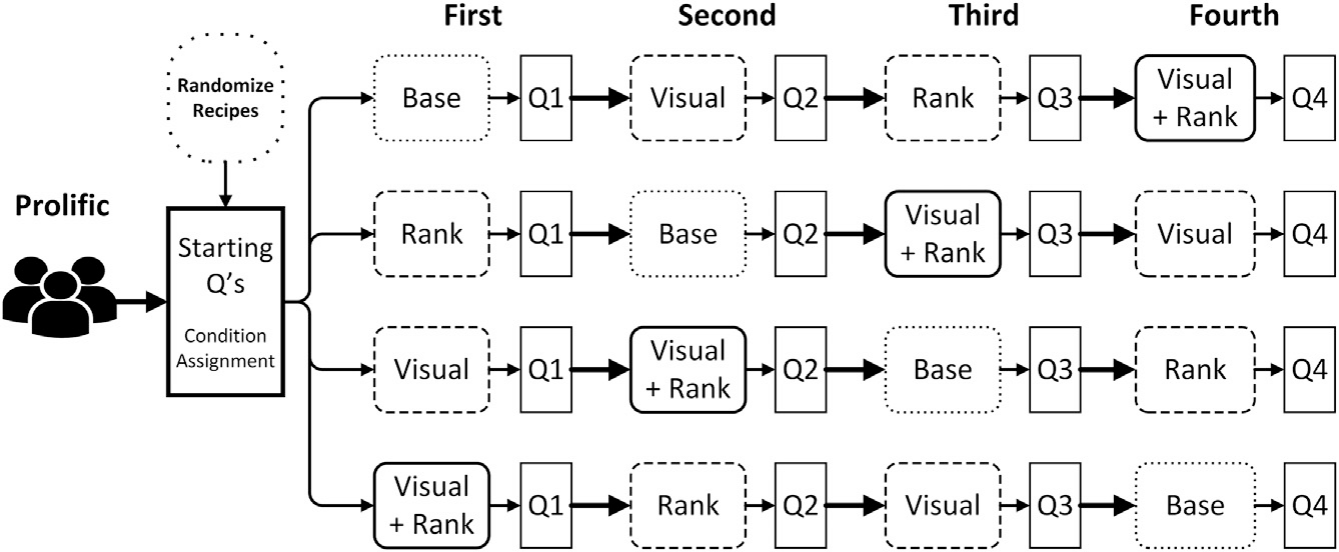
Full procedure of the current study, including the within-subject research design. After each user completed the preliminary questionnaire, they would be randomly assigned to one of four search task sequences. The queries for recipes were randomly paired with a 2 × 2-research design on how the search results were presented: either ranking recipes on their popularity (i.e., ‘Base’) or health (i.e., ‘Rank’), either matching the visual attractiveness of recipe images to health (i.e., ‘Visual’) or not, or combining both manipulations (‘Visual + Rank’). Each search task was followed by a short user experience questionnaire (e.g., ‘Q1’).

Using the prototype depicted in Figure 4, we performed an online user study to examine whether visual attractiveness and health re-ranking can support healthy food choices. The full procedure, along with the research design is depicted in Figure 5. After each participant completed a questionnaire on their current eating habits, they were randomly assigned one out of four sequences of search tasks. Users were then asked to submit a query in the search box (i.e., either “Pasta”, “Curry”, “Salad” or “Burger”), that produced a list of eight different recipes, from which they had to choose the one they liked the most.^5^ As shown in Figure 5, each choice task was followed by a short questionnaire about the attractiveness of the presented recipes (i.e., perceived list attractiveness) and the chosen recipe (i.e., choice satisfaction).

The presentation of the search results in Figure 4 was subject to a 2 × 2-within subjects design. Recipes in the baseline condition (i.e., ‘Base’ in Figure 5) were ranked on their popularity rating on AllRecipes.com (i.e., ‘Mean Rating’ in Table 3), and depicted the original database images. In contrast, the ‘Rank’ condition produced a list of recipes that was ranked on FSA score (cf. Table 3), along with the original images. In contrast, we replaced the database images in the ‘Visual’ condition. Ten out of sixteen photos of healthy recipes (i.e., a FSA score of 7 or lower) were replaced with more attractive images, while all sixteen photos of unhealthy recipes were made less attractive. Nonetheless, the search results were ordered on a recipe’s popularity rating. Finally, in the ‘Visual + Rank’ condition, we not only replaced the images, but also ordered the search results on their healthiness. This produced a list of search results of which the first four recipes were the healthiest and depicted an attractive image, while the bottom four recipes were less healthy and were accompanied by an unattractive image.

### 2.6 Participants

We determined an appropriate sample size by performing an a-priori power analysis in GPower 3.1 (Faul et al., 2009), using “F tests—ANOVA: Repeated measures, with a within-between interaction”. Under a power of 0.9, a small to medium effect size f=0.15, *α* = 0.05, and a somewhat conservative correlation among four repeated measures (0.3), our design required a minimum sample size of 160. We sampled more participants to safeguard against order effects, since they could have theoretically reduced the number of usable trials, but this was not the case.

In total, 239 participants (*M*_*age*_ = 32.2 years, *SD*_age_ = 10.8) fully completed our study. 33.3% of participants identified as male, and 98.5% finished at least high school, while 55.2% attained at least a Bachelor’s degree. Participants were recruited *via* Prolific, a British participant recruitment service that reportedly yields comparatively high data quality (Peer et al., 2017). Fluency in English was required to be included in the experiment, and participants were asked not to join the study if they were on a vegetarian or vegan diet. However, neither requirements were checked before the study. Furthermore, participants received €1.72 (₤1.50) as compensation for their participation in a single 7–9 min session.

### 2.7 Measures

#### 2.7.1 Objective Aspects

The main variable under investigation was the FSA score of the chosen recipe, which represented its healthiness. We used this score to examine whether different search result representations led to changes in the healthiness of the chosen recipe. Features underlying this score describe a recipe’s nutritional content, namely its fat, saturated fat, sugar, and salt content—all per 100 g. Furthermore, following the computational approach in Trattner et al. (2018), we also extracted low level features of recipe images and used these in our models: an image’s brightness levels, its sharpness and colorfulness, entropy [i.e., how much information needed to encode an image by a compression algorithm (Trattner et al., 2018; Thum, 1984)], and saturation (i.e., the colorfulness in proportion to its brightness). Finally, we also used the mean rating described in Table 3, which were given by users on AllRecipes.com (Trattner et al., 2018).

#### 2.7.2 User Characteristics

We asked users to report any dietary restrictions they would have. For example, vegetarianism, gluten-free, lactose intolerance, or halal. Although vegetarian or vegan participants were discouraged from participating in our study invitation, we did not correctly record responses of vegetarianism during the study due to a technical error. Besides this, we also asked users to indicate their self-reported health on a 5-point scale (i.e., very unhealthy to very healthy), as well their level of cooking expertise (i.e., whether someone often cooks at home). Finally, we inquired on a user’s age, gender, and level of education, but omitted the latter two in our analyses due to missing data.

#### 2.7.3 List Characteristics

We also considered the position of recipes in the lists of search results. We encoded a variable ‘Position in List’ from 1 to 8, where 1 represents recipes which were presented first in a list.

#### 2.7.4 User Evaluation Aspects

To address [H4–H5], we inquired on a user’s evaluation of the presented search results in each trial using short questionnaires. Users were presented eight propositions and were asked to indicate on a 5-point Likert scale to what extent they agreed with each proposition. These items were designed to capture a user’s perceived attractiveness of a list of recipes [PLA; items were based on earlier work in Willemsen et al. (2016)], as well as a user’s level of satisfaction with the chosen recipe [CS; items were based on earlier work in Willemsen et al. (2016), Starke et al., (2017), Starke et al. (2020)]. All items were submitted to a confirmatory factor analysis (CFA), as part of a Structural Equation Model analysis, with which we checked both discriminant and convergent validity.

Table 4 lists the questionnaire items and factor loadings. In contrast with findings in Willemsen et al. (2016), we could not reliably differentiate between PLA and CS, due to a violation of discriminant validity [cf. Knijnenburg and Willemsen (2015) for computational details]. Eventually, we considered a single evaluation aspect that comprised items from both PLA and CS, which will be used to assess hypotheses [H4–H5] instead of through the separate aspects. This single aspect, labelled ‘User Satisfaction’, was inferred reliably, for its content validity was high (*α* = 0.9) and its convergent validity met the requirements: the average variance explained (*AVE*) was 0.72, higher than the required 0.5.

**TABLE 4:** Factor loadings of the confirmatory factor analysis.

Item for Aspect ‘User Satisfaction’	Loading
PLA-1: The list of recipes was attractive	0.874
PLA-2: The list of recipes showed too many bad items	
PLA-3: I did not like the list of recipes	–0.849
PLA-4: The presented recipes matched my preferences	0.883
CS-1: My chosen recipe could become one of my favorites	0.813
CS-2: I would recommend the chosen recipe to others	
CS-3: I think I would enjoy eating the chosen recipe	0.801
CS-4: The recipe I chose matched my preferences	

The items on Perceived List Attractiveness (‘PLA’) and Choice Satisfaction (‘CS’) formed a single evaluation aspect: ‘User Satisfaction’. Items in grey were omitted from the final analysis, as it significantly improved the model fit. The internal consistency of the items was high: α=0.90, while the Average Variance Explained met the requirements: AVE = 0.72.

## 3 Results

We examined to what extent visual enhancement and health re-ranking of the search results led to healthier choices, without decreasing user satisfaction. First, we found evidence for our hypotheses [H1–H3], as our visual and re-ranking manipulations increased the objective healthiness of chosen recipes [H1–H3]. We contextualized these findings by exploring what types of predictors could best predict user choices: user characteristics (e.g., self-reported health), visual features, or contextual features (i.e., position in a recipe list). In a similar fashion, we addressed [H4–H5] by showing that the manipulations of our research design did not affect a user’s level of satisfaction. Finally, we explored whether we could model which recipe is chosen from the larger list of eight recipes, based on the underlying features.

### 3.1 Healthiness of Chosen Recipe

#### 3.1.1 Hypothesis Testing

To examine hypotheses [H1–H3], a two-way repeated measures ANOVA was conducted to compare the influence of visual attractiveness and health re-ranking on the healthiness of recipes chosen across four different trials. The results of the ANOVA run on 239 participants are reported in Table 5. 

**TABLE 5:** Results of a 2 × 2-Repeated Measures ANOVA on the FSA score of chosen recipes (four per user).

	Partial SS	d*f*	Mean square	*F*	(Partial) *η* ^2^
Model (on ‘FSA’)	2972.05	717	4.15	1.32**	0.80
Visual Error	36.97 1054.52	1 238	36.97 4.43	8.34**	0.034
Ranking Error	32.40 683.10	1 238	32.40 2.87	11.29***	0.045
Visual * ranking Error (between users)	1.36 1163.69	1 238	1.36 4.89	0.43 1.55***	0.61
Residual	750.14	238	3.15		

***p < 0.001, **p < 0.01, *p < 0.05.

Regarding the main effects, the results in our ANOVA (cf. ‘Visual’ in Table 5) supported hypothesis [H1]. We found that accompanying healthy recipes with attractive images and unhealthy recipes with unattractive images decreased the FSA score of chosen recipes (*M* = 7.46, *S.E.* = 0.081), compared to recipe lists that depicted baseline images (*M* = 7.86, *S.E.* = 0.081): *F*(1,238) = 8.34, *p* < 0.01, *η* ^2^ = 0.034. Furthermore, the main effect of re-ranking recipes on their FSA score (‘Ranking’) reported in Table 5 provided support for [H2]. The chosen FSA score significantly decreased when ranking recipe lists on their FSA score (M=7.48, S.E.=0.81), instead of their popularity rating (*M* = 7.85, *S.E.* = 0.081): *F*(1,238) = 11.29, *p* < 0.001, *η* ^2^ = 0.045. In other words, users were more likely to choose healthier recipes when facing a list of search results that was ordered on health, rather than popularity.

**FIGURE 6:**
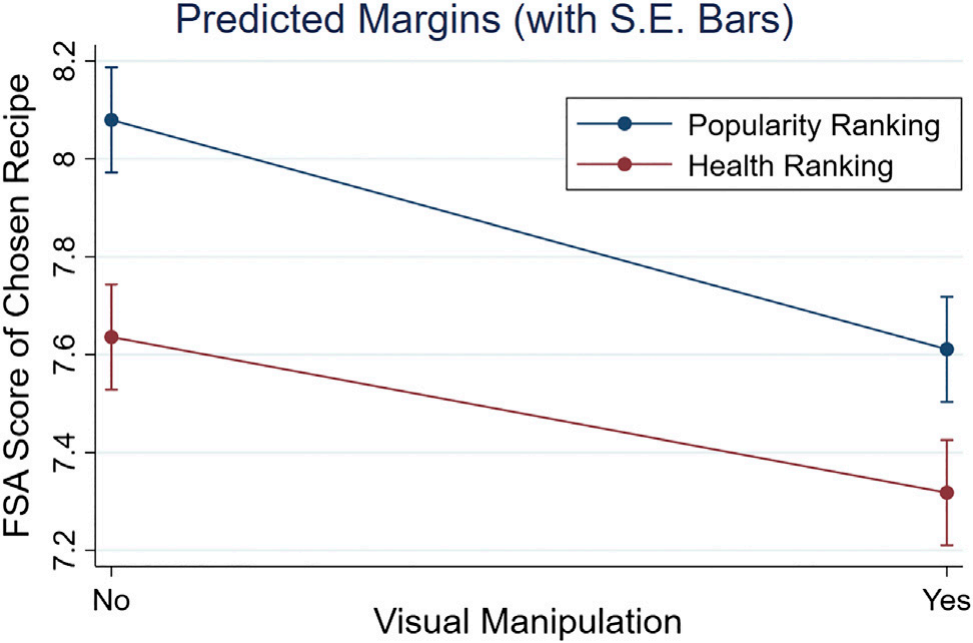
Marginal effects plot of the healthiness (FSA score) of chosen recipes per condition. The mean FSA score of recipes chosen in the baseline (i.e., Popular Ranking, no Visual Manipulation) was higher (i.e., unhealthier) than for recipe lists with a Visual Manipulation, Health Re-ranking, or both.

Finally, we expected an additive effect of the visual and re-ranking manipulations on a recipe’s chosen healthiness [H3]. As reported in Table 5, we found no significant interaction between both manipulations (i.e., ‘Visual * Ranking’): *F*(1,238) = 0.43, *p* = 0.51,^6^ supporting [H3]. Further evidence for an additive effect is depicted in Figure 6. Although the slopes of both lines were not exactly parallel, it was clear that the chosen FSA score was further lowered when combining the health re-ranking and the visual manipulation: *M* = 7.32, *S.E.* = 0.11, compared to only applying a health re-ranking (*M* = 7.64, *S.E.* = 0.11) or a visual manipulation (*M* = 7.61, *S.E.* = 0.11).

#### 3.1.2 Modelling the Healthiness of Chosen Recipes

We also explored to what extent other features and user characteristics predicted the chosen FSA score. We modelled features that were underlying our health re-ranking (i.e., position in list) and visual manipulation (i.e., image features) conditions, and examined what types of features were the best predictors: list features, recipe (image) features, or user characteristics. Since features on nutritional content (e.g., fat per 100 g) were used to calculate the FSA score, we excluded them to avoid overfitting the model.

We performed two linear regression analyses, which are reported in Table 6.^7^ We discerned between an ‘General Model’, which examined effects across all conditions, and a ‘Condition-specific Model’ that included meaningful interactions. For example, although the effect of an image’s entropy (i.e., a visual feature) on the chosen FSA was 0 across all conditions, it had a negative effect in visual conditions (i.e., ‘Entropy × Visual’ in the Condition-specific Model, β=−1.16, *p* < 0.001), compared to a non-significant effect in the baseline (i.e., ‘Entropy’). Both models in Table 6 attained good to high values of model accuracy: $R^{2}=47.6\%$ for the General Model, and $R^{2}=66.1\%$ for the Condition-specific Model. Although each model explained well why recipes were chosen in terms of their healthiness, that Condition-specific Model was the most accurate and was deemed the most representative.

**TABLE 6:** Two linear regression models predicting the FSA score of chosen recipes. Both models are divided into list and recipe features, image features, and user characteristics, including user satisfaction.

	General model		Condition-specific model	
	*β (S.E.)*	Partial *η* ^2^	*β (S.E.)*	Partial *η* ^2^
Condition–Main effects				
Health Re-Ranking			–3.04 (0.18)***	0.24
Visual manipulation			7.49 (1.75)***	0.019
List and recipe features				
Position in list (1 = top)	0.21 (0.022)***	0.094	0.22 (0.018)***	0.14
Position × Re-Ranking			0.60 (0.035)***	0.24
Popularity (rating)	–0.40 (0.053)***	0.058	–0.51 (0.044)***	0.13
Image features				
Brightness	–1.77 (0.59)**	0.0097	–1.12 (0.60)	
Brightness × visual			3.27 (1.18)**	0.0082
Colorfulness	–14.04 (0.93)***	0.20	–10.53 (0.89)***	0.13
Colorfulness × visual			–3.59 (1.73)*	0.0046
Entropy	–0.017 (0.12)		–0.17 (0.10)	
Entropy × visual			–1.16 (0.10)***	0.034
Sharpness	–4.52 (0.75)***	0.038	–1.53 (0.63)*	0.0063
Sharpness × visual			–3.58 (1.18)**	0.0097
Saturation	6.98 (0.70)***	0.098	4.60 (0.76)***	0.038
Saturation × visual			2.08 (1.51)	
User characteristics				
Male (gender)	–0.001 (0.10)		–0.0028 (0.0035)	
Cooking experience	–0.017 (0.067)		0.038 (0.053)	
Self-reported health	–0.12 (0.064)		–0.058 (0.052)	
User satisfaction	0.0048 (0.052)		–0.026 (0.041)	
Constant	12.23 (1.01)***		12.27 (0.90)***	
$R^{2}$	0.476***		0.661***	
RMSE	1.44		1.16	

The ‘General Model’ examines effects across all within-subject conditions (i.e., health re-ranking and visual manipulation), the ‘Condition-specific Model’ compares relevant interaction effects with condition variables (e.g., ‘Brightness × Visual’). Regression coefficients are denoted by β, S.E. is the standard error, and η^2^ denotes the partial effect size and can be considered an expression of the relative feature importance. ***p < 0.001, **p < 0.01, *p < 0.05.

To understand the relative importance of different predictors, we inspected the significance levels and the partial effect sizes (i.e., *η* ^2^) in Table 6. Both models showed that user characteristics did not explain the chosen FSA score, since neither demographics, nor the self-reported cooking experience and health were significant predictors. Furthermore, we found no relation between the chosen FSA score and a user’s level of satisfaction: *p* > 0.1, for both models.

In contrast, features that described a recipe itself, as well as features that represented list characteristics, significantly affected the chosen FSA score in both models. The partial effect sizes in Table 6 reveal that the image features in the General Model (e.g., colorfulness: $\eta^{2}=0.20$, and saturation: $\eta^{2}=0.098$) seemed to be better predictors than other recipe and list features (i.e., position in list: $\eta^{2}=0.094$, and popularity: η=0.058). In contrast, in the Condition-specific Model, image features (Sum of partial $\eta^{2}\approx 0.23$) were surpassed in terms of their predictive value by list and recipe features: Sum of partial $\eta^{2}\approx 0.51$. This showed that when controlling for differences across conditions, the predictive value of the position order effect increased, which was found to be the strongest in lists were ordered on health (i.e., ‘Position X Re-Ranking’) was stronger (β=0.60, p<0.001, $\eta^{2}=0.14$), compared to the effect in the popularity condition (β=0.22, p<0.001, $\eta^{2}=0.24$).

Furthermore, Table 6 also describes the main effects of the conditions. In line with [H2], we find a large, negative effect (i.e., partial $\eta^{2}=0.24$) of the health re-ranking condition on the chosen FSA score: β=−3.04, p<0.001. This indicated that the ranking condition, in addition to the aforementioned position order effects, increased the healthiness of chosen recipes. In contrast with the ANOVA reported earlier, Table 6 shows that the visual manipulation had a small, positive effect (i.e., partial $\eta^{2}=0.019$) on the chosen FSA score: β=7.49, p<0.001. Although this provided evidence against our hypothesis [H1], it seemed that the other image features in the Condition-specific Model with larger effect sizes (Sum of partial $\eta^{2}\approx 0.23$) had captured the effect of visual attractiveness.

With regard to these image features, Table 6 shows that most effects were consistent with were consistent with our visual attractiveness model (cf. Table 2) and our intended visual manipulation. Either or both models in Table 6 show that higher levels of brightness (in the General Model), colorfulness, entropy, and sharpness led to lower chosen FSA scores, while higher saturation levels led to higher chosen FSA scores. The evidence for brightness was somewhat mixed, for it negatively affected the chosen FSA score in the General Model (*β*=–1.77, *p* < 0.01), but positively affected that score in the visual condition (i.e., ‘Brightness X Visual’) in the Condition-specific Model: β=3.27, *p* < 0.01. However, the effect size was minor ($\eta^{2}=0.0082)$ compared to other image features. For one, while entropy did not affect the FSA score in the General Model, it positively affected the FSA score in the Condition-specific Model in the Visual condition (i.e., ‘Entropy × Visual’): β=−1.16, *p* < 0.01. Finally, while image’s saturation level had a positive effect on the chosen FSA score in both models (*p* < 0.001), the effect was not significantly larger in the Visual condition. Taken together, Table 6 points out which images features contributed to an image’s visual attractiveness that affected the chosen FSA score and with what magnitude.

### 3.2 User Satisfaction

#### 3.2.1 Hypothesis Testing

To examine hypotheses [H4] and [H5], we conducted a two-way repeated measures ANOVA, examining whether our visual attractiveness and health re-ranking manipulations did not decrease a user’s satisfaction level across four search tasks. The user satisfaction aspect was obtained using a confirmatory factor analysis, for which the items and factor loadings are reported in Table 4. Although we initially formulated hypotheses based on perceived list attractiveness and not user satisfaction, we considered the hypotheses using the latter aspect. Table 7 shows the results of the 2 × 2-ANOVA, which indicated that there is no significant interaction between a visually manipulated list and a list ranked on health on the user’s satisfaction: F(1,238)=2.31, p=0.13.^8^

**TABLE 7:** Results of a 2 × 2-Repeated measures ANOVA predicting a user’s satisfaction level of a presented recipe list, across four different search tasks.

	Partial SS	d*f*	Mean square	*F*	(Partial) *η* ^2^
Model (on User Satisfaction)	692.79	717	0.97	1.74***	0.84
Visual	0.28	1	0.28	0.42	
Error	162.35	238	0.68		
Ranking	0.14	1	0.14	0.21	
Error	155.97	238	0.66		
Visual * ranking	1.28	1	1.28	2.31	
Error (between users)	372.77	238	1.57	2.82***	0.74
Residual	1.45	238	0.0061		

The model’s high η^2^ is caused by User Satisfaction being strongly user-dependent. ***p < 0.001, **p < 0.01, *p < 0.05.

The ANOVA main effects in Table 7 are neither significant. In line with hypothesis [H4], we found no difference in user satisfaction between lists of recipes that were ranked on their FSA score (M=−0.021, S.E.=0.034), and lists that were ranked on their popularity rating (M=0.0034, S.E.=0.034): F(1,238)=0.65, p=0.65. This suggested that a user’s level of satisfaction did not decrease due to changing how recipes were ranked. Furthermore, we assessed whether user satisfaction did not significantly drop due to our visual manipulation. In line with [H5], we found no differences between lists that depicted visually (un)attractive images for (un)healthy recipes (M=−0.026, S.E.=0.034) and the baseline lists (M=0.0087, S.E.=0.034): F(1,238)=0.42, p=0.52, which suggested a user’s level of satisfaction was unaffected by our visual manipulations.

**FIGURE 7:**
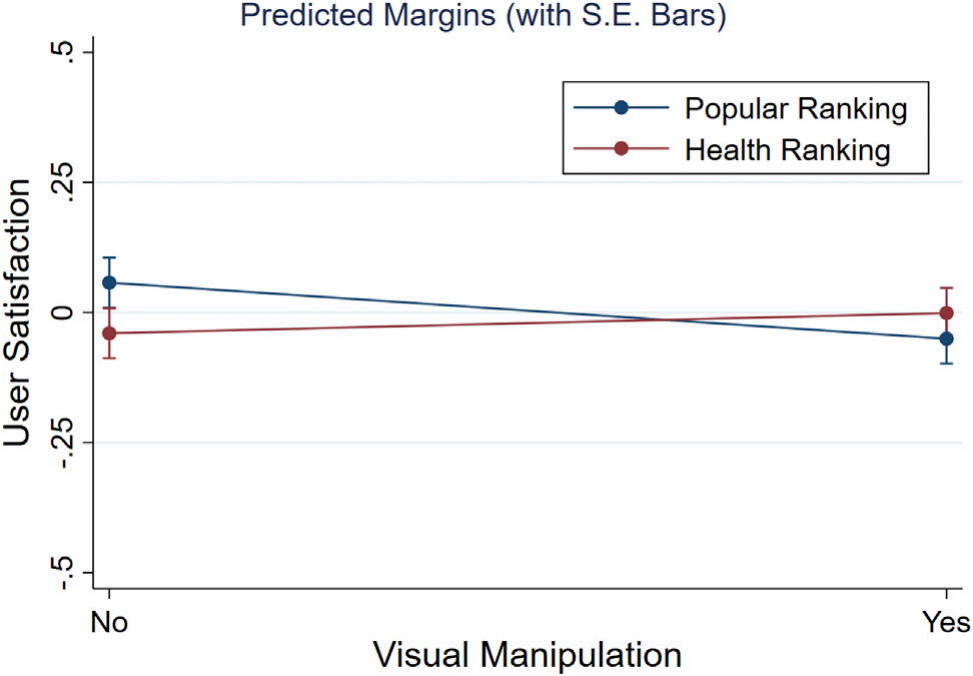
Marginal effects on User Satisfaction across our 2 × 2-within subjects design (i.e., Popular vs Health Ranking, images visually manipulated or not).

On top of [H4] and [H5], we neither expected changes in user satisfaction if both the health re-ranking and visual manipulation were combined. Besides not finding a significant interaction (cf. Table 7: ‘Visual * Ranking’), we inspected the marginal effects of the four conditions on the user’s satisfaction level for any changes. Figure 7 depicts that the reported user satisfaction is almost identical across all conditions (e.g., note the truncated Y-axis), suggesting no further combined effects of our ranking and visual manipulations. Finally, whereas the ANOVA provided no evidence for a difference across conditions, we performed equivalence tests to check whether both main effects were actually absent and not just undetected [cf. Lakens et al. (2018)]. Using two one-sided t-tests in Stata for each main effect (Dinno, 2017), we obtained evidence for absence of two main effects (both *p* < 0.001), confirming [H4] and [H5] that user satisfaction was unaffected by the visual and health ranking manipulations.

#### 3.2.2 Modelling User Satisfaction

We also tried to model a user’s level of satisfaction through various recipe and list features, as well as user characteristics. However, we could not form a significant multilevel regression model (i.e., it did not pass the Wald $\chi^{2}$ test), as the model’s *R* ^2^ was too low (3%), even when including all features.^9^ To further explore this, we computed the correlation between the chosen FSA score and the user satisfaction, but found no significant relation: r(956)=−0.017, *p* = 0.50. This supported our findings that we can support healthier food choices without decreasing a user’s level of satisfaction.

### 3.3 Predicting Choice Behavior in a List of Recipes

Finally, we sought to predict which recipe would be chosen among a list of eight alternatives, based on recipe features and list characteristics. To this end, we performed two conditional logistic regression analyses on the chosen recipes per list, omitting user characteristics for they were constant per list. Table 8 presents two models, one ‘Full Model’ and one ‘Simplified Model’, which were both clustered at the user level. Each model discerned between three categories of variables: list and recipe features, recipe healthiness, and image features. The Full Model, which broke down the healthiness (e.g., fat per 100 g) and visual attractiveness (e.g., Brightness) into lower level features, had a slightly higher pseudo *R* ^2^ (0.053 compared to 0.016).

**TABLE 8:** Two conditional logistic regression models, clustered at the user level, using recipe and list feature to predict what recipe is chosen.

	Full model		Simplified model	
	*β*	Robust S.E.	*β*	Robust S.E.
List and recipe features				
Position in list (1 = top)	−0.098***	0.017	−0.083***	0.018
Popularity (rating)	−0.12**	0.044	−0.072*	0.034
Recipe healthiness				
FSA score			0.17***	0.023
Fat/100 g	−0.069***	0.0083		
Saturated Fat/100 g	0.34***	0.041		
Salt/100 g	0.22	0.13		
Sugar/100 g	0.049*	0.019		
Image features				
Visual attractiveness			0.22***	0.054
Brightness	2.11***	0.47		
Colorfulness	−0.65	0.76		
Entropy	0.57***	0.084		
Sharpness	−0.79	0.61		
Saturation	0.094	0.59		
Pseudo *R* ^2^	0.0533***		0.0158***	

The Full Model considers all variables, while the Simplified Model uses the FSA score to represent health and Visual Attractiveness to represent the visual features. ***p < 0.001, **p < 0.01, *p < 0.05.

We discuss Table 8 from top to bottom. First, in line with our previous analyses, it showed that if a recipe was ranked higher up in a list of search results, it was more likely to be chosen: *p* < 0.001, for both models. In contrast, a recipe’s popularity was found to have a negative influence on the probability that it was chosen: *p* < 0.001, for both models. This arguably surprising outcome. was attributed to popular recipes being ranked first in the two popularity ranking conditions,^10^ which was captured by ‘Position in List’. On the other hand, popular recipes were also somewhat unhealthier on average [see also Trattner et al. (2017)], suffering from the negative modifications in our three treatment conditions.

With regard to a recipe’s healthiness, we found that unhealthy recipes were more likely to be chosen. Recipes with higher FSA scores (i.e., unhealthier ones), as well as recipes with higher levels of saturated fat (*p* < 0.001) and sugar (*p* < 0.05) were more likely to be chosen. However, unsaturated fat decreased this probability (*p* < 0.001, in the Full Model). Finally, Table 8 confirms our earlier findings that recipes with visually attractive images were more likely to be chosen (*p* < 0.001), which was attributed in this model to an image’s brightness and entropy level (both: *p* < 0.001).

## 4 Discussion

Popular internet recipes tend to be high in specific nutrients, such as fat and sodium (Trattner et al., 2018). Unfortunately, the high prevalence of popular recipes in food search interfaces reinforces unhealthy eating habits among its users. To alleviate this, we have investigated whether changing the visual attractiveness of recipe images, as well as by ranking recipes on their FSA score can support healthy food choices, without decreasing user satisfaction. This paper describes as one the first in the field of (food) information retrieval how search results should be presented, rather than optimizing what items (i.e., recipes) should be retrieved [cf. Helmy et al. (2015), Ghenai et al. (2020)]. Moreover, it shows how visual attractiveness can be modelled, as well as used to steer user preferences.

One of the merits of the study is that it presents both simple and elaborate models, of which the findings can be generalized to other applications and domains. Our hypothesis testing through straightforward ANOVAs points out that two factors can be used to empower users in their food decision-making. Through a simple re-rank on health (i.e., a recipe’s FSA score) and a model-based adaptation of recipe images in terms of their visual attractiveness (i.e., through our preliminary study), we present two ways in which the presentation of search results could be re-designed to support healthier eating habits. These effects are also confirmed in a more elaborate linear regression model, which shows that user characteristics are far less important in determining food decisions than recipe features (in our study: visual attractiveness) and position effects (in our study: a health re-ranking).

Our results have implications for research on healthy eating and digital food nudges [cf. Mirsch et al. (2017)]. In our introduction, we have discussed three types of nudges [related to cognition, affect, and behavior (Cadario and Chandon, 2020)], which can support healthy food decisions. Although it is advocated that users and customers should be educated to adopt healthier eating habits [i.e., cognitively-oriented nudges (Cadario and Chandon, 2020)], such as done by policy makers through traffic light food labelling [cf. Figure 1 (of Health UK and Agency, 2016)], it seems that users are more likely to be persuaded by affectively-oriented nudges (e.g., through visual attractiveness of food images), as well as through behaviorally-oriented nudges (e.g., position in a list). These types of nudges have been examined in ‘offline contexts’ (Kallbekken and Sælen, 2013; Bergeron et al., 2019), as well as in a couple of online user experiments (Elsweiler et al., 2017; Ghenai et al., 2020). However, our study is the first in which both recipe retrieval and food nudges are combined in a search interface, which can serve as the starting point for more elaborate work. For example, whereas we have only employed a non-personalized autocompletion approach, digital nudges could also be combined with algorithms that can prioritize a user’s interest in healthy food (Musto et al., 2020).

### 4.1 Main Contributions

With regard to visual attractiveness, this study’s contribution is two-fold. First, in our main study, we have highlighted how an image’s visual attractiveness affects user preferences, as healthier recipes were more likely to be chosen when accompanied by a visually attractive photo. This has been shown by comparing choices across conditions, as well as by modelling the relation between the underlying image features (e.g., brightness, colorfulness) and the chosen FSA score. We have in particular expanded knowledge on online recipe selection, for we have tested this principle in an actual search task with recipes lists, rather than in a choice experiment with recipe pairs, such as done by Elsweiler et al. (2017). Second, our preliminary study shows how various image features relate to visual attractiveness. It is among the first to specifically relate these features to human judgements of visual attractiveness. In doing so, we have confirmed findings that image features can predict an image’s attractiveness or popularity (Khosla et al., 2014), as well as that users are biased towards visually attractive food images (Elsweiler et al., 2017). Although there is room to improve the accuracy of our visual attractiveness model, it can be employed in future research to steer user preferences, for example, as an affectively-oriented food nudge (Cadario and Chandon, 2020).

Furthermore, our ‘post-retrieval’ health re-ranking is also a novel contribution to the field of IR. We have reported strong effect sizes for the presentation order effect on the chosen FSA score. This is consistent with literature from psychology that described how individuals favor items that are positioned first in a list (Mantonakis et al., 2009; Carney and Banaji, 2012). Whereas studies on restaurant menus show a so-called ‘edge effect’ [i.e., options listed at the top and bottom of a list are chosen more often (Bar-Hillel, 2015)], we find no evidence for ‘bottom of list’ preferences. Instead, the principle of ‘first come, first served’ seems to apply to the online selection of recipes.

Although a presentation order effect seems straightforward, it is actually often overlooked in studies where users evaluate AI. Most preferences of users, or consumers for that matter, are rather context-dependent (Bettman et al., 1998), and can be steered by small adaptations in the choice environment or architecture (Thaler and Sunstein, 2009; Johnson et al., 2012). Algorithmic approaches to food personalization assume that user preferences are rather rigid (Freyne and Berkovsky, 2010; Trattner and Elsweiler, 2017a), while in reality a user might also be receptive to slightly ‘less relevant’ search results [cf. McNee et al. (2006)]. The fact that we have found no differences in user satisfaction across conditions is a testament to this.

### 4.2 Limitations

There might be some concerns about the user evaluation aspects. We have not been able to differentiate between Perceived List Attractiveness (PLA) and Choice Satisfaction (CS), as is done in Willemsen et al. (2016), but we have used a single aspect labelled ‘User Satisfaction’. Although this has contrasted with our hypotheses, we have been able to reliably infer a single user evaluation aspect that captured items related to both PLA and CS. Hence, it is reasonable that possible detrimental effects on a user’s evaluation of a list of search results, as well as the chosen recipe, are also measured by this aspect.

We have faced issues in recording user responses about users having vegan or vegetarian eating habits. Although this could have confounded our results because of the use of meat-based recipes (cf. Table 3), we have found that controlling for any dietary restriction in our models does not significantly impact our results. Moreover, we have reported large partial effects for two of our main predictors (i.e., visual attractiveness and position in list), which are unlikely to be the result of variations in eating habits.

Furthermore, it is possible that our online search prototype (cf. Figure 4) does not fully reflect the eating habits of users, for it only offers a small number of search results (i.e., 32 recipes), across just four recipe types (i.e., burgers, curries, pastas, and salads). For example, if a user had to look up a pasta dish but dislikes that type of food, she would be inherently less satisfied with all options. Furthermore, it is also possible that the larger AllRecipes.com database contains recipes that are very unhealthy, but are also very popular (Trattner et al., 2017). This could result in users ignoring possible health gains from other recipes, by favoring such a popular recipe when looking for a meal to cook. However, we do not believe that this would have made a significant impact, as the average rating of the recipes used in this study is 4.4 stars, indicating that our recipes are still relatively popular. Moreover, we wish to emphasize that our results show no differences in user satisfaction across all conditions. Nonetheless, we definitely advocate to validate our results in a larger study in which users are offered a larger set of recipes to choose from.

It could be argued that some order effects occurred because users expected the recipe lists to employ a popularity-based ranking, as done on most websites (Trattner et al., 2018). Although one could argue that the effectiveness of a health re-ranking would be reduced if users are explicitly notified about this, earlier research has shown that nudges can still work when users are informed about them (Loewenstein et al., 2015). Moreover, we have not observed any differences in user satisfaction across conditions for users with varying levels of self-reported health, which could play a role when inspecting a recipe list. In fact, most personalized recipe lists, such as in food recommender systems, are optimized for relevance (Trattner and Elsweiler, 2017a), which may end to be health-based rather than popularity-based.

Finally, we have focused in our study on main dishes. It is possible that a health re-ranking might be less effective or even lead to a reduction of user satisfaction for other dish types. For example, research on knowledge-based food recommender systems shows that different eating goal features determine choices for desserts, compared to main dishes (Musto et al., 2020). We would encourage future contributions to also look into other meal types, as well as other recipe keywords to further examine the effectiveness of health re-ranking and visual attractiveness in supporting healthy food choices.

### 4.3 Future Research

One interesting avenue of future research is to develop a more sophistical model of visual attractiveness. While this paper uses a parametric linear regression, non-parametric machine learning could be used to optimize model performance, as well as to learn more complex, latent image features. Moreover, the visual attractiveness manipulation in the current study is based upon manual selection and manipulation of existing images. Although all the selection and adaptations of database images have met our set requirements, it would be interesting to develop an approach in which this can be done automatically.

We wish to emphasize that the lessons learned in this study could be used beyond existing food retrieval systems and diversified to other domains, For example, the use of a ranking strategy that is not popularity-based can also be applied to other features than health, such as by re-ranking of list of photo cameras in a web shop on price or zoom capabilities. Cross-domain use of visual attractiveness models is also possible, but this should be done cautiously, for it might require more ethical considerations. For example, would it be desirable for web shops to promote specific products with the goal of maximizing profits, by means of changing how they are presented visually? Since related techniques are already being applied, for example, Netflix personalizes preview images for TV series to match a user’s genre preferences (Gomez-Uribe and Hunt, 2015), it should be an interesting topic for future research to examine the ethical implications of visual adaptations.

Furthermore, this study has focused on online food choices. It would be very useful and interesting to gain a better understanding of how such online choices translate to actual behavior. For example, previous research in energy conservation suggests that HCI can support short-term behavioral change, as well as that higher levels of user satisfaction increase the likelihood that online choices are put into practice (Starke et al., 2017). However, there are few studies on dietary interventions that are assisted by technology or AI. Hence, it would be interesting to monitor the health of users of a recipe application similar to ours over a longer time period.

To conclude, this study is interdisciplinary in nature. It uses approaches from fields, such as Information Retrieval and Behavioral Economics, to improve the state-of-the-art in Food AI. We feel that using such a broad scope is fundamental to face challenges on healthy eating. We encourage the deployment of more studies in which algorithmic optimization is combined with digital nudges and rigorous user evaluation.

## Data Availability

The datasets presented in this study can be found in the following online repository: https://github.com/alainstarke/RecipeSearch.

## Footnotes

1 The values for these features were obtained using the OpenIMAJ Java Framework, v1.3.5: http://www.openimaj.org. Please refer to Trattner et al. (2018) for more details.

2 Entropy refers here to the average amount of information that is coded per image (dot) (Thum, 1984), which is usually higher for ‘focused’ images.

3
https://lucene.apache.org/

4
https://twitter.github.io/typeahead.js

5 Although larger lists of search results are common on some websites, we wished to avoid choice overload effects [cf. Scheibehenne et al. (2010)] that might further reinforce users to pick the first option, which is a possible consequence of showing many items (Bollen et al., 2010).

6 Analyses in this subsection were also performed without users who reported to have an allergy (*N* = 37), but this did not significantly affect the results.

7 Although the structure of our dataset, which consisted of four trials per user, would warrant a multilevel regression analysis, the clustering coefficient ρ for both models was zero. This meant that a regular regression was sufficient.

8 Similar to the analyses performed in the previous subsection, excluding users who reported to have an allergy did not significantly affect the results.

9 Note: Most of the variance of user satisfaction was found to be clustered at the user level, which had likely caused the high *η* ^2^ values for the model reported in Table 7.

10 We checked whether the popularity rating’s direction of effect was caused by the presence of other predictors in the model, but the *β*-value was unaffected by the removal of other predictors.
